# A theory of the neural mechanisms underlying negative cognitive bias in major depression

**DOI:** 10.3389/fpsyt.2024.1348474

**Published:** 2024-03-12

**Authors:** Yuyue Jiang

**Affiliations:** University of California, Santa Barbara, Santa Barbara, CA, United States

**Keywords:** major depressive disorder, cognitive bias, limbic system and emotion, frontal-limbic network, neurobiology, cognitive dysfunction, cognitive impairment

## Abstract

The widely acknowledged cognitive theory of depression, developed by Aaron Beck, focused on biased information processing that emphasizes the negative aspects of affective and conceptual information. Current attempts to discover the neurological mechanism underlying such cognitive and affective bias have successfully identified various brain regions associated with severally biased functions such as emotion, attention, rumination, and inhibition control. However, the neurobiological mechanisms of how individuals in depression develop this selective processing toward negative is still under question. This paper introduces a neurological framework centered around the frontal-limbic circuit, specifically analyzing and synthesizing the activity and functional connectivity within the amygdala, hippocampus, and medial prefrontal cortex. Firstly, a possible explanation of how the positive feedback loop contributes to the persistent hyperactivity of the amygdala in depression at an automatic level is established. Building upon this, two hypotheses are presented: hypothesis 1 revolves around the bidirectional amygdalohippocampal projection facilitating the amplification of negative emotions and memories while concurrently contributing to the impediment of the retrieval of opposing information in the hippocampus attractor network. Hypothesis 2 highlights the involvement of the ventromedial prefrontal cortex in the establishment of a negative cognitive framework through the generalization of conceptual and emotional information in conjunction with the amygdala and hippocampus. The primary objective of this study is to improve and complement existing pathological models of depression, pushing the frontiers of current understanding in neuroscience of affective disorders, and eventually contributing to successful recovery from the debilitating affective disorders.

## 1 Introduction

Globally, more than 280 million people of all ages suffer from depression (1). The economic burden of major depressive disorder (MDD) among US adults mounted to $US326 billion in 2018 (year 2020 values); the share attributable to workplace costs increased from 48 to 61% (2). Therefore, addressing depression is an urgent matter that requires collective efforts from both society and the government. Although the etiology of depression has received extensive attention and scientific input in recent years, its pathogenesis remains unclear. This is mainly due to the complicated nature of the etiology of depression involving mechanisms of various aspects such as environmental, genetic, cognitive, and neurological influences (3).

The cognitive theory of depression, first postulated by Aaron Beck, posits that the vulnerability to depression stems from negatively biased errors in thinking (4), which may include an individual’s thoughts, inferences, attitudes, and interpretations, and the way in which they attend to and recall information. (5). At the same time, several common depressogenic thinking errors are presented (6, 7), among which most involve a pattern of emphasis on the negativity focusing on the self, the environment, or the future. Some errors included are catastrophizing (making pessimistic predictions about the future without substantial evidence), labeling (self-classification in a negative light following an adverse event), mental filtering (concentration on negative information while dismissing positive information), and overgeneralization (assuming that one negative event signifies a trend of more unfortunate occurrences) (8). These cognitive errors tend to affect automatic thoughts and biases in attention, interpretation, and memory (9), and their diminishing consequences on the information processing system are widely observed in patients with depression.

In fact, there’s growing evidence that Depression is characterized by an emphasis on negative information in information processing (5) and implicated in its etiology and treatment (10, 11). Clinically, Cognitive biases in depression have been studied in the context of abnormalities in attention, interpretation, and memory processes, especially for negative information, as well as cognitive control impairments (12). Studies involving individuals experiencing various stages of depression, including dysphoric states, clinical depression, and those in remission from depression, have consistently demonstrated a distinct inclination toward directing attention to negative information. This bias is notably more pronounced when compared to nondepressed samples (13). Furthermore, clinical observations have provided empirical support for the idea that individuals with depression, as well as those with a history of depressive episodes, face difficulties in effectively ignoring and disengaging from emotionally negative stimuli that are evidently unrelated to the task at hand (14). These findings support the pervasive impact of cognitive biases on individuals with depression. The role of memory bias in depression has also been documented. Depression is closely linked to explicit memory biases, where individuals experiencing depression tend to exhibit a propensity to recall more over-general and negative memories while recalling fewer specific and positive memories when compared to nondepressed individuals (15, 16).

Numerous neuroscience studies have provided substantial evidence of brain damage and neuroplastic changes in depression, shedding light on their potential contributions to observed cognitive errors. Dysfunctions in neural circuits involving the prefrontal cortex, thalamus, temporal cortex, striatum, and limbic system have been implicated in the symptomatology of depression (17, 18). Disner and his colleagues, as well as Beck himself, have provided a comprehensive review of the cognitive deficits and their corresponding brain regions in depression. The cognitive bias, including Biased attention, Biased processing of emotional stimuli, Biased thoughts and rumination, and Biased memory processes, has been found to involve the brain areas of ventrolateral prefrontal cortex (VLPFC), dorsolateral prefrontal cortex (DLPFC), the amygdala, the hippocampus, medial prefrontal cortex (MPFC), the rostral anterior cingulate cortex (ACC) activity (19). One noteworthy finding is the volumetric reduction observed in the hippocampus among patients with depression (20). Additionally, Ghosal et al. (21) have pinpointed GABAergic deficits and circuit dysfunction in the prefrontal cortex as characteristic of this mood disorder. Moreover, an imbalance in activity within specific regions of the prefrontal cortex has been observed, characterized by hyperactivity in the ventromedial prefrontal cortex (vmPFC) and hypoactivity in the dorsolateral prefrontal cortex (dlPFC) (22). These altered neurocircuits have the potential to influence the information processing of individuals with depression, leading to heightened negative recollection, quicker responses to sad language, and a more pessimistic interpretation of ambiguous phrases or situations (23).

Nevertheless, while researchers have successfully described neural mechanisms and their association with depression, they fall short of elucidating why these mechanisms specifically contribute to a bias favoring negative processing (19). Despite the extensive body of research on the neuromechanics of depression, brain damage, and the cognitive symptoms and deficits in depression, particularly concerning information processing patterns related to negative cognition, there is a dearth of studies that comprehensively bridge these two levels and propose how neuro mechanisms contribute to cognitive deficits. This absence of comprehensive integration leaves a gap in understanding how these mechanisms ultimately lead to information processing patterns that prioritize negative perceptions.

**Figure 1:**
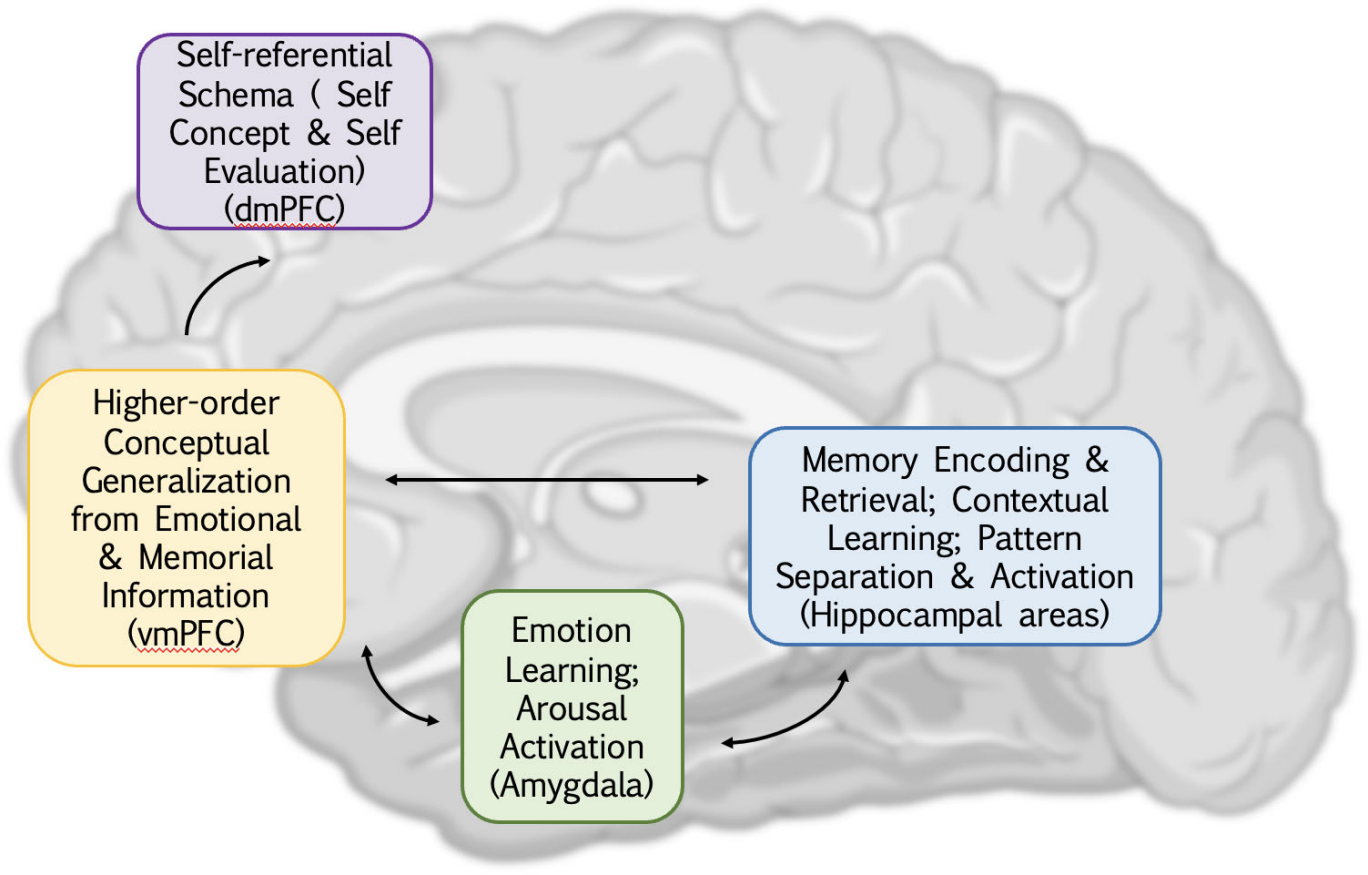
An overview of cognitive dysfunction in depression and its corresponding brain regions. The abnormal functional connectivity of the amygdala, hippocampus, and ventral medial prefrontal cortex (vmPFC) illustrates the mutual influence between emotions, memory, and higher-level generalization, contributing to a negative processing system in depression and a significant driver of negative cognitive bias. Additionally, the integration of vmPFC and dorsal medial prefrontal cortex (dmPFC) extends this information, introducing biases not only toward the external world but also toward oneself. This biased thinking intensifies the reception of negative information, establishing a closed loop.

In response to this gap, this paper introduces a neural model that underlying the cognitive bias favoring negative information in depression, predominantly involves interactions within corticolimbic systems (see **Figure 1**). The interaction of the amygdala, hippocampus, and PFC is studied in terms of their activity level and functional connectivity. We propose that the excessive activity of the amygdala, potentially driven by associative learning from negative stimuli, results in an automatic cognitive bias toward negative information and impairs the capacity to experience positive emotions. Simultaneously, the hippocampus reinforces the recollection of mood-congruent memories and hinders the extraction of positive memories through emotional activation generated in the amygdala. While the amygdala initiates the stress response through the hypothalamic-pituitary-adrenal (HPA) axis, it further complicates this relationship, adversely affecting the pyramidal neurons in the hippocampus, as chronic stress alters its structure and exacerbates depressive symptoms. The ventromedial prefrontal cortex (vmPFC) assesses the emotional stimulus through stimuli-response learning thus potentially resulting in an exaggeration of negative emotions. Conversely, when the vmPFC interacts with the hippocampus in inference-based memory retrieval, it encourages the creation of an extensive memory framework centered on negativity. This recall and reinterpret could affect the emotional valance of stored memories, emphasizing their negative emotional aspects. This can lead individuals to develop a cognitive bias favoring a negative perspective of the external world. Over time, this process may lead to exaggerated negative thoughts and biases in their perception of the environment and subjective experiences, ultimately culminating in a pervasive negative cognitive bias.

## 2 Positive feedback regulation of amygdalar activation allows excessive negative emotional responses

Research has shown that individuals’ bias to interpret ambiguous stimuli negatively tends to operate at an automatic level (see comments by 9 and 24). The amygdala is thought to be involved in and primarily responsible for emotional processing, especially those caused by and associated with fear and threat (25). Data from studies examining the human amygdala indicate that a substantial proportion of its neurons exhibit heightened responses to unpleasant stimuli, in contrast to a relatively limited response to pleasant stimuli (26). This suggests that the amygdala plays a pivotal role in shaping the facets of negative emotion.

The basolateral amygdaloid complex (BLA), a subsection of the amygdala, has been found to receive input from various sensory modes in sensory processing areas of the cortex (27–29). Various sensory inputs, encompassing visual, auditory, and tactile information originating from external stimuli or internal thoughts and memories, are integrated within the amygdala (30). Subsequently, this information is directed toward the central nucleus of the amygdala (CEA), enabling the amygdala to swiftly and automatically assess how to respond, often preceding conscious awareness. Based on this automatic assessment, the amygdala activates different pathways for emotional responses (31). The CEA has been described to serve as the amygdala’s interface with fear-response systems (32). Hyperactivity in the amygdala is a major pathological factor in depression (33, 34), which may contribute to the emotional processing that comprises the cognitive bias favoring negative information. It also plays a role in the overactivation of fear and anxiety-related circuits, impairing the ability of other cortex regions to inhibit the fear responses induced by the amygdala (35). Considering this as a positive feedback loop: a hyperactive amygdala tends to exert greater inhibition on the inhibitory functions of other brain regions, particularly the cortex and hippocampus. This reciprocal loss of inhibition contributes to heightened amygdalar activation. The amygdala persists in an excited state, lacking an effective mechanism for termination. Consequently, individuals with depression may find it challenging to experience positive emotions or inhibit negative emotional responses when due to these neural dynamics.

Heightened amygdala activity in individuals with depression may lead to increased associative learning in response to negative stimuli that would strengthen the association between these stimuli and negative emotions (36). Many hypotheses suggest that this associative learning in the amygdala is facilitated through the mechanism of Long-Term Potentiation (LTP) (37–39). Within the amygdala, this potentiation primarily occurs at synapses responsible for transmitting information related to threatening stimuli. Through LTP, the amygdala establishes a robust link between sensory cues associated with a threat and the ensuing emotional and physiological responses, such as fear and stress. Furthermore, drawing from Pavlov’s principles of learning, it is worth noting that LTP can be induced in initially “weak” synaptic pathways if the activity in these pathways is paired with activity in a pre-existing “strong” pathway (32). In terms of cognitive bias, this suggests that when individuals encounter unfamiliar stimuli that elicit negative emotions, the associative learning-induced plasticity may trigger the strengthening of previously enhanced pathways, promoting learned association with negative emotions in the new stimuli. This process can consequently result in generalized negative emotions in response to ambiguous stimuli. The prolonged hyperactivity of the amygdala in depression is sustained due to the absence of external inhibition from other brain regions, coupled with internal long-term potentiation (LTP) promotion. The hyperactivity of the amygdala forms a robust basis for the subsequent hypotheses in this paper.

## 3 Hypothesis 1: bidirectional amygdalohippocampal projection activate negative emotion-memory Loop

In brain imaging of patients with depression, both the amygdala (40–43) hyperactivity and hippocampal dysfunction (44–46) was discovered and confirmed by a large number of studies. The anatomical structure of the hippocampus comprises three main components, including the Dentate Gyrus, the subdivision CA1-CA4, And the striatum which is responsible for the output of hippocampal information (47). In processing information, the hippocampus draws mainly from the entorhinal cortex, mossy fibers originating from the dentate gyrus (DG) provide sparse and powerful excitatory connections to CA3 PCs (48). These connections are proposed to assist in the encoding of new patterns of activity (representing new memories) in CA3 through pattern separation (49). The CA3 region of the hippocampus has been ascribed a pivotal role both in forming associations during encoding and in reconstructing a memory representation based on partial cues during retrieval (50). This is thought to require plastic changes in the strength of specific synaptic contacts (51).

The negative emotion from the amygdala can extend its influence through interaction with various regions of the brain. This extensive connectivity of the amygdala to other brain regions impacts many cognitive functions (52). Notably, the activation of the amygdala, particularly the basolateral amygdala (BLA), has been shown to exert a modulating effect on plasticity in the hippocampus (53), especially involving the consolidation of memories of emotionally arousing experiences (54, 55). On the neuronal level, BLA achieves this by the establishment of monosynaptic and glutamatergic circuits to the ventral CA1 in the hippocampus (56). Research has found that such projections, given high contingency, would be able to Trigger heterosynaptic LTP at Hippocampus-To-Accumbens Synapses, which allows increased reinforcement of emotionally charged episodic memory (57). In addition, the stimulation of the basolateral amygdala (BLA) has been observed to induce long-term potentiation (LTP) in the dentate gyrus (DG) (58), which can lead to memory enhancement due to emotional enhancement (59). In depressed individuals, however, as discussed earlier regarding the excessive activity of the amygdala in depression and its emphasis on negative information, it can be predicted that the amygdala will be able to send contingent negative emotion-related projections into the hippocampus. This LTP induced by such projections in the hippocampus may be a factor in the biased memory for negative stimuli observed in depression.

On the other hand, impaired activity in the DG/CA3, as well as in the lateral CA1, was found to be associated with depressive symptoms, even at a subclinical level (60). In the context of depression, the connection between the amygdala and the paraventricular hypothalamus, whether through direct or indirect pathways, triggers the release of adrenocorticotropic hormone (ACTH) from the pituitary gland (61). ACTH subsequently circulates to the adrenal glands situated atop the kidneys, prompting the adrenal cortex to release glucocorticoids (CORT). CORT’s impact on the hippocampus is multifaceted. In mildly stressful situations, low or moderate levels of circulating CORT enhance explicit memory formation by acting on the hippocampus, a phenomenon referred to as the hippocampal negative feedback loop (62). However, elevated levels of circulating CORT, typically associated with chronic stress, can disrupt the physiological functioning of the hippocampus, resulting in the dysregulation of its glucocorticoid system (63). This surge in glucocorticoids can harm the glucocorticoid receptors (GR) within the hippocampus and have downstream effects on NMDA receptors (64), ultimately leading to the dysregulation of the hypothalamic-pituitary-adrenal (HPA) axis (65). This dysregulation is often associated with early-stage reversible dendritic remodeling in pyramidal granule neurons within the CA1 and CA3 regions, along with parallel reversible changes in synaptic terminal structures. Simultaneously, the loss of pyramidal neuronal dendrites in both the CA3 and CA1 regions has been observed in patients with major depressive disorder (MDD) (63).

The hippocampus region CA1-CA4 has been described to have to property of an attractor network (66). This “attraction” of network activity to a stored pattern provides a useful form of associative memory (67). This property is mainly due to The extensive excitatory interconnections between CA3 pyramidal cells, in which associative memories are stored and recalled through pattern completion (68, 69). pyramidal dendrites enable the occurrence of distinct activity patterns (multistability) that have been associated with different items stored in memory (70) Any of these patterns can be activated by a partial input since the recurrent connections amplify and thereby complete the initial activation pattern (71). Due to the crucial role pyramidal cells play in establishing and maintaining connections within the hippocampal attractor network, their loss is likely to disrupt the hippocampus’s ability to simultaneously activate distinct patterns of activity within the attractor network. This disruption could then result in a reduction in the associativity and comprehensiveness of memories, leading to memory fragmentation and difficulties in fully retrieving and integrating memories. Furthermore, the impairment of the brain’s capacity to recognize patterns and associations can hinder the ability to relate different pieces of information and experiences, ultimately diminishing cognitive flexibility. In essence, this disruption in the hippocampal network can result in difficulties in memory processing, memory integration, and cognitive adaptability.

Yet it’s crucial to recognize that the reduction in pyramidal cells may not follow a linear pattern. In a 21-day induced depression experiment on an animal model, dynamic alterations in CA1-3 pyramidal cells were observed. Specifically, on day 14 (metaphase), certain depression-like behaviors manifested, concomitant with the inhibition of basal synaptic transmission and an enhancement of Long-Term Potentiation (LTP) at CA3-CA1 synapses. However, when assessed on day 21, a different pattern emerged. LTP induction was impaired, and the basal synaptic transmission at hippocampal CA3-CA1 synapses was diminished. This was accompanied by a reduction in dendritic spines among CA1 and CA3 pyramidal neurons. Furthermore, there was a simultaneous decrease in the levels of brain-derived neurotrophic factor (BDNF) within the hippocampus (72).

**Figure 2:**
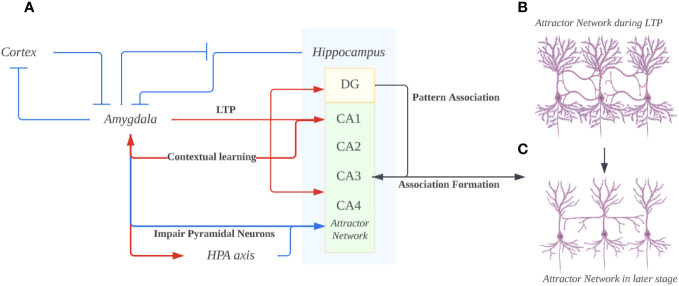
**(A)** The overall bidirectional interaction of Amygdala and Hippocampus. The hyperactivity of the amygdala prevents suppression in itself from the cortex and hippocampus. Through projections, the amygdala induces long-term potentiation (LTP) in the hippocampus’s Dentate gyrus (DG), CA1, and CA3. Concurrently, it adversely affects the hippocampus, particularly pyramidal neurons, by excessively activating the hypothalamic-pituitary-adrenal (HPA) axis. The DG utilizes a pattern association code to encode memories, transmitting this code to CA3, thereby facilitating memory consolidation. Ultimately, the hippocampus provides contextual cues to the amygdala, fostering contextual learning and exacerbating the amygdala’s hyperactivity. **(B)** The hippocampus attractor network in the early stage of depression. Due to the hyperactive amygdala enhancing the LTP in both DG and CA1/3 regions, the pyramidal neurons would increase in arborization and form more connections. According to the mood-congruent memory theory, the synapse that’s enhanced/formed would be closely related to negatively charged emotions that are pervasive in the amygdala. **(C)** The hippocampus attractor network in the long-term depression. The pyramidal neurons dendrites under the impairing effects from hyper-activated amygdala projections. Such loss of dendrites would lead to the dysfunction of memory retrieval, especially the hardship of memories involving happy or positively charged emotions since unlike the negatively-charged memories, they aren’t enhanced in the first place. (Created with BioRender.com).

Based on these findings, it is plausible to suggest that during the early stages of depression, the hippocampus undergoes an initial phase of heightened connectivity among pyramidal cells (see **Figure 2B**). In this phase, memories associated with negative emotions become consistently activated in the hippocampus, primarily driven by the substantial influx of negative emotional input from the amygdala, which strengthens the neural connections between sensory information related to threats and the ensuing emotional and physiological responses. Consequently, the hippocampus adapts to this heightened arousal of negative emotions by establishing additional synaptic connections, thus providing an explanation for the observed enhancement of Long-Term Potentiation (LTP) at CA3-CA1 synapses. During this early stage, the hippocampus appears to be in the process of learning to adapt to the dominance of negative emotions in memory activation. It achieves this adaptation by increasing the synaptic weight associated with negative memory arousal through LTP mechanisms. On the cognitive level, this aligns with the concept of mood-congruent memory, wherein memories with a negative emotional charge are more frequently recalled in a negative emotional context (73). This heightened recall of negative memories can lead to the long-term reinforcement of negative emotion-related memories and, therefore, contribute to the initial development of a cognitive bias schema in depression (74). Simultaneously, the continuous stimulation of the amygdala causes ongoing damage to the hippocampus during its hyperactivity phase by overactivating the HPA axis (75). This persistent stimulation ultimately results in a decline in pyramidal neurons due to a deficiency in neurotrophic factors over the long term (76). As we previously discussed, the decline in pyramidal neurons not only leads to difficulties in forming new memories but also severely impairs hippocampal connectivity, significantly compromising overall hippocampal function (see **Figure 2C**).

Due to the increased synaptic weight of previous negative memory arousal, coupled with the difficulty in establishing new synaptic connections due to the decline of pyramidal neurons, the increasing arousal of negative emotions in the hippocampus, as well as the difficulty in non-negative emotion arousal. Through the direct projection, the amygdala will induce LTP primarily associated with negative emotional content. Such effect from the amygdala may prevent the possibility of other positive/neutral memories entering the negative emotion-memory cycle, whereas a person may have difficulty forming positive memories and related memory cues due to difficulty retrieving memories associated with positive emotions (77). This in turn creates a closed loop of storage and retrieval for repressing positive emotions. These two effects, combined, may cause individuals to fall into negative emotion-memory cycles more frequently. Negative emotions reinforce negative emotional memories while suppressing memories and experiences of positive emotions. This leads to more negative emotions, creating a vicious cycle.

Reversely, Projections from the hippocampal formation to the amygdala have been shown to have a potential influence on contextual learning in amygdala. (78, 79). Ventral CA1 (vCA1) hippocampal neurons encode and convey contextual representations through monosynaptic projections to the amygdala. The contextual information is then integrated with aversive signals in the amygdala for fear memory formation. Strengthening of the hippocampal–amygdala pathway as a consequence of learning can facilitate the activation of the amygdala, resulting in conditioned fear responses to the threat-predictive context during the recall of contextual fear memory6 he projection from the ventral hippocampus to the prefrontal cortex and then to the amygdaloid basal nuclei has been found to be active during fear renewal. Simultaneously, the disconnection of the ventral hippocampus from the basal nuclei impairs fear memory renewal (78).

In our hypothesis, the hippocampus, by providing various contextual cues to the amygdala (through activation of different sensory cortices), can activate the reexperience or replay of events (80). Considering the enhanced processing of negative information mentioned earlier, the hippocampus can provide the amygdala with numerous cues related to negative memories. Due to this interaction, the amygdala provides affective stimuli to the hippocampus to assist in the encoding and retrieval of negatively charged memories. During retrieval, the hippocampus activates synapses established in the past, once again stimulating the amygdala to respond to negative emotions. This response may potentially activate the HPA axis, damaging the dendritic network of the hippocampus and making the retrieval of other neutral or positively charged memories more challenging. In this way, the cyclic interaction between the hippocampus and the amygdala continuously rehearses and enhances the same negative information, memories, and emotions, making it difficult for individuals to escape this cognitive trap of negativity (see **Figure 2** for a summary).

## 4 Hypothesis 2: vmPFC generalizes information from amygdala and hippocampus in higher-level negativity cognitive processing

While the automatic level of information processing plays a crucial role in depression, it’s important to recognize that cortical control of these automatic processing systems can also exert a significant influence (81, 82). This cortical management serves to monitor and adjust the output of heuristic processing, including the default mode (83, 84).

In the context of depression, a common symptom involves a deficiency in cortical control over the limbic system, particularly in emotional regulation. Such deficiencies may stem from the omission or improper weighting of pertinent information and the interference of irrelevant information, leading to decision-making errors or biases (85, 86), and are primarily served by the prefrontal cortex (87).

Hyperactivity of the ventromedial prefrontal cortex (vmPFC) has been consistently observed in patients with depression (22, 88–90). The precise function of the vmPFC has been the subject of ongoing debate, with two main hypotheses currently accepted. The first hypothesis suggests that the vmPFC plays a role in emotion regulation by inhibiting the activity of the amygdala (91, 92). The second hypothesis proposes that the vmPFC is involved in generalizing emotional responses and guiding decision-making processes (93). Interestingly, there is evidence supporting both seemingly conflicting hypotheses. In this paper, we favor the second hypothesis for several compelling reasons. First and foremost, a significant body of fMRI studies has consistently reported hyperactivity in both the ventromedial prefrontal cortex (vmPFC) and the amygdala among patients with depression. If the vmPFC were primarily responsible for inhibiting amygdala function, these concurrent hyperactivities would present a paradoxical scenario. Second, the existing evidence supporting the inhibitory role of the vmPFC on amygdala activity predominantly stems from studies involving healthy individuals (94, 95). Notably, no studies have successfully demonstrated this inhibitory effect of the vmPFC on amygdala activity in patients with depression. This suggests the possibility that in the brains of individuals with depression, the vmPFC may lack the capacity for effective inhibition. Taken together, these reasons lead us to favor the hypothesis that the vmPFC’s primary function in depression lies in the generalization of emotional responses and the guidance of decision-making processes, rather than in inhibitory control over the amygdala.

Considerable data from various studies point to the ventromedial prefrontal cortex (vmPFC) playing a role in encoding information related to reinforcement outcomes (96–98). Functional imaging data further reveal that resting activity in the vmPFC is correlated with the subjective experience of negative affect (99). This implies that the vmPFC may have a significant role in generating emotional responses. One possible explanation for the vmPFC’s involvement in generating emotional responses lies in its dense connections to the basolateral and central nuclei of the amygdala, as well as to visceromotor structures such as the hypothalamus and periaqueductal gray (100, 101). Additionally, studies have shown that damage to the vmPFC reduces the expected strengthening activity of the basolateral amygdala, providing further support for this hypothesis (102).

VmPFC plays a pivotal role in guiding higher-order reinforcement learning by integrating and interpreting emotional information, which subsequently informs decision-making processes (98, 103, 104). vmPFC appears to receive reinforcement expectation information in stimulus–reinforcement-based learning and thus may be responsible for encoding reinforcement outcome information (96, 98, 105). Indeed, some studies even suggest that the vmPFC serves as a representation of value information. In a state-based model, the vmPFC is postulated to be involved in the initial encoding and establishment of an abstract-state space, essentially serving as a benchmark for value judgment. Standard reinforcement learning (RL) mechanisms are then employed to learn and adapt the values associated with these abstract states within the model (106, 107). Consequently, the vmPFC is likely to contribute to the enhanced and biased representation of the world following the integration of emotional information from the hyperactive amygdala. This heightened amygdala activation can lead to biased cognitive judgments of the individual regarding the external environment, leveraging extensive associative learning of negative emotions and enhancement processes in the amygdala.

Meanwhile, the role of the ventromedial prefrontal cortex (vmPFC) in memory generalization should not be overlooked. In the context of episodic memory, it has been demonstrated that generalized memory representations can arise from the integration of information across multiple events, a process mediated by interactions between the vmPFC and the hippocampus (108). This process of memory generalization holds significance as it may underpin the formation of certain cognitive judgments. Importantly, the interplay between the hippocampus and vmPFC may have significant implications for the valence of the stored memory, particularly in the context of concept learning and generalization. This role of the vmPFC aligns with findings from studies on episodic memory, demonstrating that the vmPFC supports the integration of current experiences with prior knowledge (109, 110). This promotes the retention of pattern-consistent information (111) and facilitates new relational inferences across overlapping events (109, 112). Therefore, the way the memory is retrieved may bias the nature of concept representations formed during learning and accessed during generalization (108).

Consequently, an individual with hyperactive ventromedial prefrontal cortex (vmPFC), amygdala, and hippocampus may perceive the external environment as fearful, dangerous, and unwelcoming. This perception arises through a sequence of processes. First, the amygdala is more prone to stimulation, responding with heightened negative arousal. Subsequently, the vmPFC integrates and further assesses this emotional response, potentially leading to an overgeneralization of the negative emotional reaction. On one hand, the hippocampus is more inclined to process memories associated with negative emotions, primarily due to the amygdala’s influence. On the other hand, it is likely that when the vmPFC interacts with the hippocampus, it fosters the creation of generalized memory representations predominantly centered around negative emotions. Consequently, individuals may develop a cognitive bias that leans toward the external world being characterized by negativity. Through this retrieval and processing of memory, individuals may redefine the emotional valence of their memories, accentuating their negative emotion-related attributes. Over time, this can lead to exaggerated negative thoughts and biased perception of the environment and subjective experience fostering a pervasive negative cognitive bias, leading individuals to perceive the external environment as fearful and terrifying.

Simultaneously, the interplay between the hippocampus, vmPFC, and dmPFC likely contributes to the development of negative self-orientation in individuals with depression, a concept introduced by Beck in 1976. Both the vmPFC and dmPFC are interconnected brain regions known to play roles in self-awareness and self-reflection (113). Previous lesion studies have associated damage to the vmPFC with a loss of self-insight (114). Furthermore, such damage has been linked to a marked reduction in certain types of negative affect, particularly emotions like shame, guilt, embarrassment, and regret (115). Notably, these emotions all involve an element of self-awareness or self-reflection.

The dmPFC is active during tasks that require individuals to contemplate themselves, their characteristics, and their preferences (116, 117). This self-referential processing is fundamental for fostering self-awareness and self-reflection. While the vmPFC contributes to the emotional awareness of self, the dmPFC processes self-relevant information (118). The collaboration between these regions aids individuals in making sense of their emotional reactions within the context of their beliefs, values, and past experiences.

**Figure 3:**
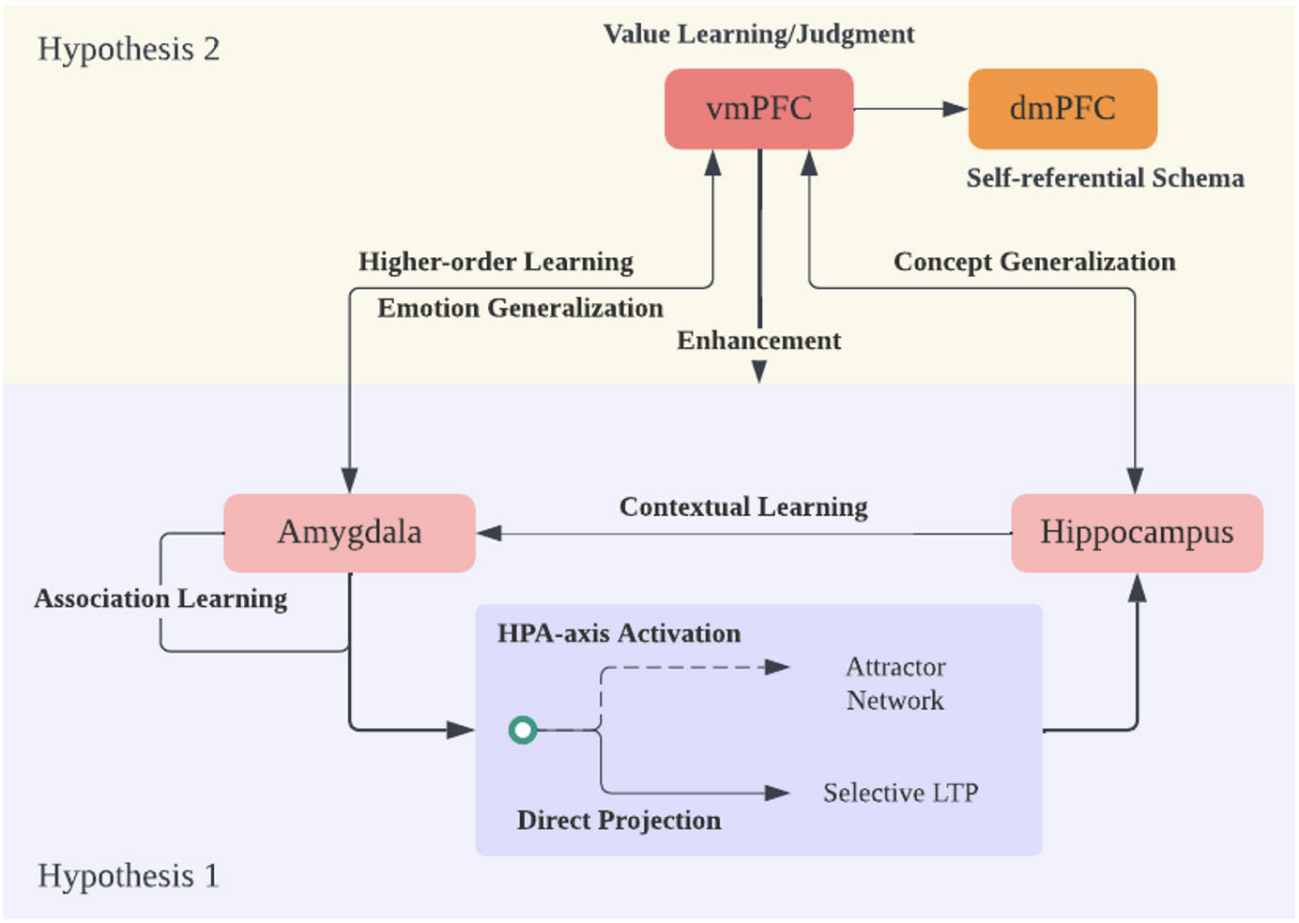
Hypothesis 2 and Theory Overview. Expanding on hypothesis 1 regarding the amygdala and hippocampus interaction, the ventral medial prefrontal cortex (vmPFC) plays a crucial role in generalizing emotional and memory/knowledge information into a broader context. The vmPFC achieves this by evaluating the world through a potential reinforcement learning mechanism. It engages in emotion and concept generalization, constructing a cognitive framework or schema focused on negativity due to its amplification of negative emotions and memories. Additionally, it performs higher-order learning of amygdala information, enhancing amygdala sensitivity. Finally, the information from vmPFC and dorsal medial PFC (dmPFC), which responds to self-referential information and schema, is integrated, expanding the negative cognitive framework into the realm of self-concept.

In individuals with depression, there is evidence of hyperactivity in both the ventromedial prefrontal cortex (vmPFC) and dorsomedial prefrontal cortex (dmPFC) (119). This heightened activity contributes to the engagement of self-related information-processing mechanisms. Building upon the earlier analysis of the model, it can be anticipated that the self-related information processed in this state will predominantly manifest as negative. The emergence and activation of this negative self-evaluation have a profound impact on the individual’s self-perception. For instance, in Beck’s cognitive model of depression, a negative self-view constitutes a significant component of cognitive impairment in depression. It consistently influences the patient’s negative perspective not only of themselves but also of the external environment and their future. (see **Figure 3** for a summary).

## 5 Discussion

This paper offers a plausible account for why individuals with Major Depressive Disorder (MDD) often display a tendency to respond to and interpret ambiguous stimuli negatively, which is coined as cognitive bias toward negative (see **Figure 3** for an overview). Two key hypotheses are presented aimed at explaining the role of various brain structures in shaping cognitive biases toward negative information and emotions in depression. Our first proposal emphasizes the significant role of the amygdala in emotion regulation and highlights how associative learning from negative stimuli in the amygdala can lead to an automatic cognitive bias favoring negative information. We have put forth a hypothesis suggesting that the hippocampal attractor network exhibits a bias toward memories associated with negative emotions, influenced by the dual impact of amygdala activation. To further substantiate this hypothesis, it is imperative to gather neuroanatomical evidence that elucidates the functional connectivity between the amygdala and the hippocampus. Additionally, research should focus on understanding the potential shrinkage of pyramidal dendrites in this context, as it may be linked to a diminished capability for parallel processing and the concurrent activation of distinct memories. These investigations would provide essential insights into the mechanisms underpinning cognitive biases toward negative information and emotions. The second hypothesis posits that the ventromedial prefrontal cortex (vmPFC) shapes an extensive cognitive framework with a negative bias through concept generalization, primarily in interaction with the amygdala and hippocampus.

Numerous depression models that focus on specific brain regions have been proposed (for summary, see **Table 1**). The Amygdala Hyperactivity Hypothesis suggests that amygdala overactivity in depression may lead to mood dysfunction and heightened sensitivity to negative stimuli (120–123). Simultaneously, the Prefrontal Cortex Dysfunction Hypothesis highlights that prefrontal cortex dysfunction in depression can result in challenges regulating negative emotions, perceiving reward and punishment, cognitive control, and decision-making in general (128–130). The Hippocampal Volume Reduction Hypothesis, supported by extensive research, posits that reduced hippocampal volume observed in depression could impact accurate memory recall (124–127). More recent theories, such as the Default Mode Network Dysregulation Hypothesis, emphasize the DMN’s association with self-referential thinking and mind-wandering (131, 132). Despite successful attempts to comprehensively explain cognitive biases in depression, a more global and functionally oriented approach to elucidate the selective formation of negativity bias remains elusive (see 19). This paper critically integrates the content of the aforementioned theoretical models, identifying dysfunctions in the amygdala, hippocampus, and PFC, and providing an in-depth explanation of their bidirectional relationship with negative cognitive bias. More importantly, it seeks to connect how unbalanced or dysfunctional functional links between various brain regions manifest in the formation of depressive negativity bias in thinking or behavioral patterns. By combining multi-layered analyses from structure to function to behavior, the paper aims to explain the development of negative cognitive schemas in depression.

**Table 1:** Summary of current hypotheses and theories of depression.

Hypothesis	Key Points	Brain Regions	Depression Explained	Supporting Studies	Limitations
Amygdala Hyperactivity Hypothesis	Larger right medial subnuclei volumes in MDD, increased right volume ratios. Amygdala linked to emotional states, stress, and HPA axis activation	Amygdala	Mood dysfunction, heightened sensitivity to negative stimuli	120–123	Consider solely single brain region cannot account for comlex and multifaceted symptoms in depression
Hippocampus Hypothesis	Reduced hippocampal volume in depression, complex relationship with various factors	Hippocampus, entorhinal cortex	Impaired memory retrieval and memory inhibition	124–127	Consider solely single brain region cannot account for comlex and multifaceted symptoms in depression
Prefrontal Cortex Dysfunction Hypothesis	Dysfunction in prefrontal cortex in depression, affecting emotion regulation, decision-making;Deficits in inhibition affecting emotion regulation, hindering recovery from negative affect	ventral medial prefrontal cortex, dorsalateral prefrontal cortex, ventralateral prefrontal cortex	Difficulties in regulating negative emotions, perceiving reward and punishment, cognitive control, decision-making	128–130	explain only the cognitve aspecrs of depression symptoms; cannot account for affective aspects
Default Mode Network Dysregulation Hypothesis	Dysregulation in the Default Mode Network, associated with self-referential thinking	DMN(medial prefrontal cortex, posterior cingulate cortex, and the inferior parietal lobule)	Association with self-referential thinking, mind-wandering	131, 132	No global explanation for negativity bias formation
Emotional Material Processing Bias Hypothesis	Biased processing of emotional material, difficulties disengaging from negative material	Limbic system, Dorsolateral prefrontal cortex, Ventromedial prefrontal cortex, Anterior cingulate cortex, Medial frontal cortex	Biased processing of emotional material, difficulties disengaging from negative material	19	Few studies on emotion-regulation strategies in depression
Current theory (this paper)	Bidirectional relationships between amygdala, hippocampus, and PFC contribute to cognitive bias.	Amygdala, hippocampus, ventromedial PFC (vmPFC)	Cognitive bias toward negative information and emotions	N/A	Assumes interconnected role of multiple regions, may require empirical validation.

Notably, the two hypotheses proposed can stand alone since they are functionally independent. Hypothesis one posits bidirectional projections between the hippocampus and amygdala, which operate independently of any involvement of the vmPFC. Hypothesis two proposes that the vmPFC retrieves information from both the hippocampus and amygdala to facilitate emotional and conceptual generalization. Although the validation of hypothesis one may enhance this process by providing consistent emotional and memory input, facilitating the vmPFC’making value judgments and evaluating the external environment, it is not necessary for hypothesis 2 to be true. Even if the projections between the amygdala and hippocampus do not specifically reinforce or synchronize negative emotions and memory, the vmPFC can still generalize emotional responses from the amygdala and extract memory representations from the hippocampus for conceptual generalization. These processes are distinct from the proposed functional/neurological connections between the hippocampus and amygdala in hypothesis 1. To validate the proposed hypotheses, further studies should explore the relationship between the vmPFC and the amygdala to ascertain whether generalization of learning and emotions occurs. Additionally, it is crucial to investigate whether the roles of the vmPFC and the hippocampus in concept generalization directly impact the cognitive processes of individuals with depression, potentially resulting in distinct definitions of negativity compared to individuals without this condition. These studies would significantly contribute to our understanding of how brain structures interact to create cognitive biases in depression. On the other hand, if our assumptions are not empirically validated or are even proven false, it opens up a new perspective on cognitive biases in depression. As this paper primarily explores the connections between the cortisol-limbic system, counterevidence might demonstrate that cognitive functional issues in depression do not lie in these more foundational emotional and emotion control cortices. Instead, they may involve higher-order cortical processing beyond our current understanding.

The limitation of this model lies in its exclusive focus on certain brain areas and the omission of other regions responsible for information processing and control, such as the Anterior Cingulate Cortex (ACC), Nucleus Accumbens, Middle Temporal Gyrus, and others (19, 130). While there is no direct evidence suggesting their involvement in negativity bias, future research should explore and investigate the potential direct or indirect contributions of these brain regions to the processing of negativity bias. This exploration would contribute to a more comprehensive understanding of the neural mechanisms underlying negativity bias. Furthermore, it’s crucial to acknowledge that no universally accepted explanation for depression currently exists (133). This paper takes an approach to analyze neuropathology related to depression within the context of cognitive theory, but it may not offer a comprehensive account of the full psychopathology of depression. Since physiological differences vary among depressed patients, the proposed model may not be applicable to all individuals suffering from depression. It’s important to recognize the diversity of depressive experiences and consider multiple perspectives in the ongoing study of depression. The intricate interplays between neurobiological and cognitive factors provide a framework for understanding depression symptoms and cognitive biases, shedding light on the significant interactions among the amygdala, the hippocampus, vmPFC, and depression. Future research should focus on examining the exact nature of cognitive bias in depression and its relation to other cognitive processes. Studies should examine whether this cognitive bias involving the dysregulation of brain areas in the frontal-limbic system is primarily related to the maintenance of depressive episodes or increased risk for first onset and recurrence of episodes. One possible explanation for the episodic/recurrent nature of depression, given the content of this theory, is the increased vulnerability of depression coupled with the onset of an acute stress-predicting event. In the mid to late stages of depression, the attractor network may become impaired due to the sparsity of dendrites in pyramidal neurons, laying the groundwork for depression recurrence. Simultaneously, because the ventral medial prefrontal cortex (vmPFC) continuously adjusts its judgments of the world and values based on the hippocampus and amygdala (while the dorsal medial prefrontal cortex (dmPFC) adjusts cognitive perceptions of oneself), unless intervened, an individual’s perception of external and internal stimuli tends to become more negative. Therefore, each depressive episode increases the risk of later depression. This explains the Kindling effect in depression (134). Finally, investigating the neural and genetic factors related to cognitive dysfunction in depression can provide a more comprehensive understanding of the disorder.

## Data availability statement

The original contributions presented in the study are included in the article/supplementary material. Further inquiries can be directed to the corresponding author.

## Author contributions

YJ: Writing – original draft, Writing – review & editing.
